# Mangosteen pericarp components alleviate progression of prostatic hyperplasia and mitochondrial dysfunction in rats

**DOI:** 10.1038/s41598-019-56970-2

**Published:** 2020-01-15

**Authors:** Hui-Hsuan Tsai, Chia-Wen Chen, Pei-Ling Yu, Yu-Ling Lin, Rong-Hong Hsieh

**Affiliations:** School of Nutrition and Health Sciences, College of Nutrition, Taipei Medical University, Taipei, Taiwan

**Keywords:** Metabolomics, Prostate

## Abstract

Prostatic hyperplasia, characterized by progressive hyperplasia of glandular and stromal tissues, is the most common proliferative abnormality of the prostate in aging men. A high-fat diet (HFD) usually is a major factor inducing oxidative stress, inflammation, and an abnormal state of the prostate. Mangosteen pericarp powder (MPP) has abundant xanthones which can be antioxidant, anti-inflammatory, and antiproliferative agents. Therefore, the purpose of this study was to research whether MPP supplementation can affect the progression of prostatic hyperplasia. Twenty-four male F344 rats were randomly divided into four groups, including a control group (C), prostatic hyperplasia-induced group (P), prostatic hyperplasia-induced with low-dose MPP group (PL), and induced with high-dose MPP group (PH). The P, PL, and PH groups were given weekly intraperitoneal injections of 3,2′-dimethyl-4-aminobiphenyl (DMAB) at 25 mg/kg body weight for 10 weeks, and simultaneously fed an HFD for 24 weeks. Our findings first demonstrated that MPP consumption significantly decreased the prostate weight, serum testosterone and dihydrotestosterone concentrations, protein expression of proliferating cell nuclear antigen, and malondialdehyde levels and ameliorated mitochondrial function in prostatic tissues. These results suggest that MPP supplementation could be used to attenuate the progression of prostatic hyperplasia.

## Introduction

Prostatic hyperplasia is a common urologic disease that mostly affects elderly men, and it is classified as benign prostatic hyperplasia (BPH) or prostate cancer based on its severity. BPH, characterized by progressive hyperplasia of glandular and stromal tissues, is the most common proliferative abnormality of the prostate in aging men. Although research indicated that the prostate size is positively associated with increasing age^1^, pathologically, BPH presents as enlarged epithelial cells and stromal cells in the prostate. Prostatic hyperplasia has negative impacts on a patient’s quality of life because prostatic hyperplasia usually induces lower urinary tract symptoms, including increased frequency of urination, increased urgency of urination, painful urination, and excessive passage of urine at night^2,3^.

Several factors were suggested to be associated with prostatic hyperplasia development, including oxidative stress^4–6^, inflammatory mediators^7–9^, and androgens whose abnormally increased levels can cause increased oxidative stress and immoderate expressions of growth factors which play crucial roles in the progression of prostatic hyperplasia^10–12^. Oxidative stress is a cellular condition which occurs when there is an imbalance between the production and clearance of reactive oxygen species (ROS). In addition, mitochondrial DNA (mtDNA) mutations may inhibit oxidative phosphorylation, increase ROS generation, and thus lead to cell proliferation and tumor growth of the prostate gland^13^. The probability of mtDNA mutations in the prostate gland increase as people get older^14^. In addition, consumption of a high-fat diet (HFD) activates lymphocytes and macrophages, upregulates expressions of inflammation mediators such as inducible nitric oxide synthase (iNOS) and cyclooxygenase-2 (COX-2), and thus induces inflammation in prostate tissues^15^. HFD intake enhances ROS generation through elevated expressions of nicotinamide adenine dinucleotide phosphate (NADPH) oxidase subunits causing nuclear factor-κB (NF-κB) activation and induction of NF-κB-upregulated expressions of inflammation mediators, such as COX-2 and iNOS^16^. Those studies demonstrated that both oxidative stress and inflammation of the prostate can be activated by an HFD, which can transform the prostate gland into an abnormal state. Moreover, several studies used 3,2′-dimethyl-4-aminobiphenyl (DMAB) injections in F344 rats to induce prostate lesions to investigate the protective potency of anticarcinogenic agents and dietary nutrients^17–20^.

In recent years, increasing numbers of studies on phytochemicals have been conducted. Mangosteen (*Garcinia mangostana* Linn.), a tropical fruit, originated from the Sunda Islands of the Malay Archipelago and the Moluccas of Indonesia. As a traditional medicine, mangosteen pericarp powder (MPP) is commonly used to cure wounds and skin infections, and treat abdominal pain and diarrhea^21^. Major polyphenol compounds of MPP are xanthones. The most abundant compound among MPP xanthones is alpha-mangostin, which can be used as an antioxidant^22^, anti-inflammatory^23^, and antiproliferative agent^24^. However, few studies of MPP treatment for prostatic hyperplasia progression have been conducted.

Therefore, the purpose of this study was to research whether an MPP intervention can attenuate the progression of prostatic hyperplasia via decreasing inflammation and improving mitochondrial function in the prostate gland after DMAB injections to induce prostate lesions in F344 rats.

## Results

### Effects of MPP on food intake, caloric intake, and body weight

After 24 weeks of experimental feeding, the food intake of prostatic hyperplasia-induced groups significantly decreased compared to the C group. However, there were no significant difference between the prostatic hyperplasia-induced groups. As to caloric intake, there were no significant differences among all groups (Table 1). The body weight (BW) of the P group significantly increased compared to that of the C group. Supplementation with MPP for 24 weeks significantly decreased the BW gain of rats in the PL and PH groups by 33.0% and 36.8%, respectively, compared to the P group (Table 1). BWs and food intake levels of animals in the different groups over the course of the study are shown in the Supplementary Information Section (Supplementary Figs. 1, 2).

**Table 1:** Weight gain of the mangosteen pericarp powder (MPP) supplement groups were significantly decreased after 24 weeks of feeding.

	C	P	PL	PH
Initial weight (g)	154.9 ± 0.7	149.7 ± 4.4	154.0 ± 5.7	156.2 ± 2.4
Final weight (g)	423.7 ± 6.4	515.1 ± 9.6*^b^	398.7 ± 7.6^a^	387.3 ± 5.2^a^
Weight gain (g)	268.8 ± 5.9	365.4 ± 7.3*^b^	244.7 ± 6.7^a^	231.1 ± 6.3^a^
Food intake (g/rat/day)	17.4 ± 0.1	13.4 ± 0.3*	13.6 ± 0.5	13.9 ± 0.4
Total caloric intake (kcal/day)	69.7 ± 0.5	67.2 ± 1.3	69.2 ± 2.4	72.3 ± 2.1
Carbohydrates (kcal)	44.3 ± 0.3	22.2 ± 0.4*	22.8 ± 0.8	23.8 ± 0.7
Fat (kcal)	11.1 ± 0.1	34.2 ± 0.7*	35.2 ± 0.8	36.8 ± 1.1
Protein (kcal)	14.3 ± 0.1	10.8 ± 0.2*	11.2 ± 0.4	11.6 ± 0.3

Values are presented as the mean ± SEM, n = 6; C, control group; P, prostatic hyperplasia-induced group; PL and PH, prostatic hyperplasia-induced and supplemented with low-dose and high-dose MPP groups, respectively; *Significantly different between the C and P groups at p < 0.05; ^abc^Values in a column with different superscript letters significantly differ at p < 0.05 compared to the P group.

### Effects of MPP on organ weights

The prostate, liver, and fat weights of rats in the P group significantly increased compared to those of the C group, and percentages of the prostate weight/BW and liver weight/BW also increased (*p* < 0.05). However, MPP supplementation significantly decreased the percentages of prostate weight/BW in both the PL and PH groups by 18.9%, and the percentages of liver weight/BW significantly decreased by 26.4% and 34.2%, respectively, compared to the P group (*p* < 0.05; Table 2). Moreover, weights of the epididymis fat and perirenal fat of the PH group were the lightest among all groups (*p* < 0.05).

**Table 2:** Liver and prostate weights of the mangosteen pericarp powder (MPP) supplement groups were significantly decreased after 24 weeks of feeding.

	C	P	PL	PH
Prostate (g)	3.32 ± 0.05	4.35 ± 0.14*^b^	2.62 ± 0.16^a^	2.83 ± 0.07^a^
Heart (g)	1.49 ± 0.08	1.39 ± 0.05	1.43 ± 0.07	1.39 ± 0.03
Liver (g)	12.63 ± 0.23	22.37 ± 0.85*^c^	12.85 ± 0.75^b^	11.04 ± 0.14^a^
Kidney (g)	2.33 ± 0.08	2.57 ± 0.04	2.40 ± 0.11	2.42 ± ± 0.03
Epididymis fat (g)	9.58 ± 0.14	15.84 ± 1.29*^c^	8.76 ± 1.28^b^	4.05 ± 0.41^a^
Perirenal fat (g)	14.74 ± 0.57	19.63 ± 1.38*^c^	7.86 ± 1.64^b^	4.13 ± 0.52^a^
Prostate weight/BW	0.78 ± 0.03	0.90 ± 0.02*^b^	0.73 ± 0.02^a^	0.73 ± 0.02^a^
Heart weight/BW	0.35 ± 0.02	0.32 ± 0.02	0.32 ± 0.02	0.33 ± 0.02
Liver weight/BW	2.92 ± 0.08	4.36 ± 0.13*^c^	3.21 ± 0.13^b^	2.87 ± 0.05^a^
Kidney weight/BW	0.57 ± 0.01	0.55 ± 0.01	0.60 ± 0.03	0.60 ± 0.03

Values are presented as the mean ± SEM, n = 6; C, control group; P, prostatic hyperplasia-induced group; PL and PH, prostatic hyperplasia-induced and supplemented with low-dose and high-dose MPP groups, respectively; *Significantly different between the C and P groups at p < 0.05; ^abc^Values in a column with different superscript letters significantly differ at p < 0.05 compared to the P group.

### Effects of MPP on lipid profiles, testosterone, and dihydrotestosterone in serum

The low-density lipoprotein cholesterol (LDL-C) concentration of the P group was significantly higher, but the high-density lipoprotein cholesterol (HDL-C) concentration was significantly lower compared to those of the C group. Supplementation with MPP significantly decreased total cholesterol (TC) (12.9% and 31.6% in the PL and PH groups, respectively, *p* < 0.05), triglycerides (TGs) (49.7% and 64.9%, *p* < 0.05), and LDL-C (46.3% and 53.0%, *p* < 0.05). Moreover, supplementation with MPP significantly increased HDL-C in the PL and PH groups by 21.8% and 26.1%, respectively, compared to the P group (*p* < 0.05; Table 3). Levels of testosterone and dihydrotestosterone (DHT) in the P group were significantly higher compared to those of the C group after 24 weeks of feeding. However, MPP supplementation significantly decreased levels of testosterone (68.3% and 74.8% in the PL and PH groups, respectively) and DHT (29.5% and 42.2%, respectively) compared to the P group (*p* < 0.05; Table 3).

**Table 3:** Blood lipid profiles, and dihydrotestosterone (DHT) and testosterone levels of the mangosteen pericarp powder (MPP) supplement groups were significantly ameliorated after 24 weeks of feeding.

	C	P	PL	PH
	**Initial**			
DHT (ng/mL)	72.5 ± 2.0	69.3 ± 2.6	67.2 ± 3.3	73.3 ± ± 1.6
Testosterone (ng/mL)	0.17 ± 0.04	0.22 ± 0.04	0.19 ± 0.03	0.15 ± 0.03
TGs (mg/dL)	70.0 ± 4.6	72.3 ± 2.4	69.7 ± 1.9	65.7 ± 4.7
TC (mg/dL)	52.0 ± 1.0	51.3 ± 1.3	53.7 ± 1.0	51.8 ± 0.9
LDL-C (mg/dL)	5.7 ± 0.4	6.0 ± 0.4	6.2 ± 0.4	5.3 ± 0.3
HDL-C (mg/dL)	14.0 ± 0.4	14.3 ± 0.0	14.0 ± 0.4	13.8 ± 0.3
	**After**			
DHT (ng/mL)	101.7 ± 6.5	149.2 ± 11.1*^b^	105.2 ±15.6^a^	86.2 ± 6.4^a^
Testosterone (ng/mL)	0.52 ± 0.04	1.39 ± 0.45*^b^	0.44 ± 0.05^a^	0.35 ± 0.03^a^
TGs (mg/dL)	138.5 ± 9.3	159.0 ± 18.7^b^	80.0 ± 5.5^a^	55.8 ± 4.9^a^
TC (mg/dL)	93.3 ± 5.7	88.2 ± 5.8^b^	76.8 ± 3.0^b^	60.3 ± 2.2^a^
LDL-C (mg/dL)	5.8 ± 0.6	27.0 ± 2.2*^b^	14.5 ± 2.3^a^	12.7 ± 0.7^a^
HDL-C (mg/dL)	82.7 ± 3.7	46.8 ± 1.1*^a^	57.0 ± 1.2^b^	59.0 ± 1.0^b^

Values are presented as the mean ± SEM, *n* = 6; C, control group; P, prostatic hyperplasia-induced group; PL and PH, prostatic hyperplasia-induced and supplemented with low-dose and high-dose MPP groups, respectively; TGs, triglycerides; TC, total cholesterol; LDL-C, low-density lipoprotein cholesterol; HDL-C, high-density lipoprotein cholesterol; *Significantly different between the C and P groups at *p* < 0.05; ^abc^Values in a column with different superscript letters significantly differ at *p* < 0.05 compared to the P group.

### Effects of MPP on prostatic epithelial hyperplasia

**Figure 1:**
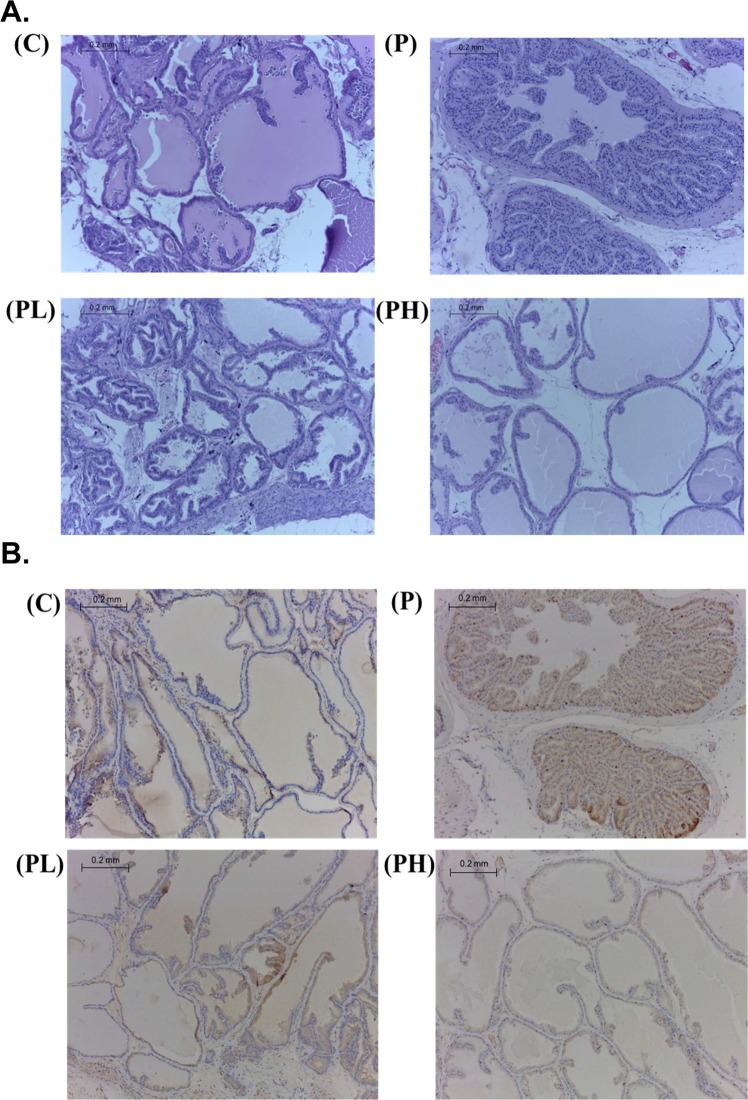
Light microscopy of prostate sections from experimental groups. Pathological changes in the prostate of rats in different groups in part A (H&E staining, 100×) and part B (immunohistochemistry of ki67, 100×). C, control group; P, prostatic hyperplasia-induced group; PL and PH, prostatic hyperplasia-induced and supplemented with low-dose and high-dose mangosteen pericarp powder (MPP) groups, respectively.

**Figure 2:**
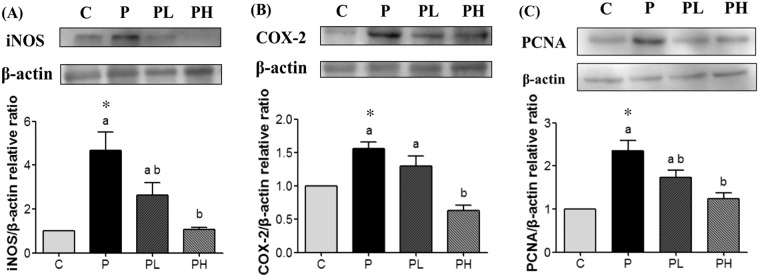
Mangosteen pericarp powder (MPP) supplementation decreased the inflammatory progress of prostatic hyperplasia. Expressions of inflammation-related proteins analyzed by Western blotting. (**A**) Inducible nitric oxide synthase (iNOS; 131 kDa). (**B**) Cyclooxygenase-2 (COX-2; 72 kDa). (**C**) Proliferating cell nuclear antigen (PCNA; 36 kDa). C, control diet; P, prostatic hyperplasia-induced group; PL and PH, prostatic hyperplasia-induced and supplemented with low-dose and high-dose MPP groups, respectively. Data were normalized to β-actin, so that the value of the control group is regarded as 1.0. Values are presented as the mean ± SEM (*n* = 6); *Significantly different between the C and P groups at *p* < 0.05. ^abc^Values in a column with different superscript letters significantly differ at *p* < 0.05 com*p*ared to the P group.

A histological analysis revealed changes in characteristics of glandular hyperplasia with epithelial proliferation in P group rats. However, MPP treatment suppressed these hyperplastic patterns (Fig. 1). In the immunohistochemical (IHC) analysis, the P group had higher ki67 expression than did the C group, and it was brownish-yellow, indicating that there was abnormal cell proliferation. In the intervention group, the ki67-stained section of the prostate tissue was smaller than that in the P group, indicating less abnormal cell proliferation (Fig. 1). This was further supported by results of proliferating cell nuclear antigen (PCNA) which showed increased protein expression in the P group compared to the C and MPP groups (Fig. 2). These results indicated that the HFD and DMAB induced prostatic epithelial hyperplasia in F344 rats, and MPP treatment could suppress abnormal cell proliferation in the prostate.

### Effects of MPP on inflammation of prostatic tissues

Inflammatory factors are crucial in the cell proliferation of prostatic hyperplasia^25–27^. As shown in Fig. 2, treatment with the HFD and DMAB significantly increased iNOS and COX‐2 protein expressions in the P group compared to the C group (*p* < 0.05). However, MPP groups exhibited significantly reduced expressions of these inflammatory proteins (*p* < 0.05). These results revealed that MPP supplementation decreased the inflammatory progression of prostatic hyperplasia.

### Effects of MPP on the mitochondrial function of prostatic tissues

**Figure 3:**
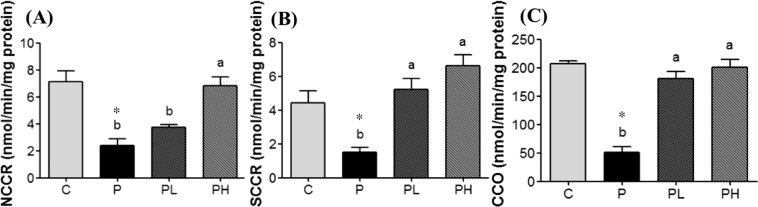
Mitochondrial enzyme activities of the prostate in the different groups after 24 weeks of mangosteen pericarp powder (MPP) treatment. (**A**) NADH-cytochrome *c* reductase (NCCR) activity, (**B**) succinate-cytochrome *c* reductase (SCCR) activity, and (**C**) cytochrome *c* oxidase (CCO) activity. Values are presented as the mean ± SEM (*n* = 6); *Significantly different between the C and P groups at *p* < 0.05. ^abc^Values in a column with different superscript letters significantly differ at *p* < 0.05 com*p*ared to the P group; C, control group; P, prostatic hyperplasia-induced group; PL and PH, prostatic hyperplasia-induced and supplemented with low-dose and high-dose MPP groups, respectively.

Nicotinamide adenine dinucleotide-cytochrome c reductase (NCCR), succinate-cytochrome c reductase (SCCR), and cytochrome c oxidase (CCO) activities of the P group were all significantly lower compared to those of the C group (*p* < 0.05), which revealed that mitochondrial function in the prostate was downregulated in rats with prostatic hyperplasia. However, MPP treatment significantly increased activities of mitochondrial complex enzymes of rats in the PL and PH groups compared to the P group (*p* < 0.05; Fig. 3). Thus, the results demonstrated that MPP supplementation might improve the mitochondrial function of the prostate.

### Effects of MPP on the oxidative stress of prostatic tissues

Activities of catalase (CAT), glutathione peroxidase (GPx), and glutathione reductase (GRd), and the reduced glutathione (GSH) content in the P group were all significantly lower compared to those of the C group (*p* < 0.05; Table 4). However, MPP treatment significantly increased GPx activity compared to the P group (*p* < 0.05). The MDA level of the P group was significantly higher compared to the C group (*p* < 0.05). However, MDA levels of the PL and PH groups were significantly reduced by 53.5% and 58.3%, respectively, compared to the P group (*p* < 0.05; Table 4).

**Table 4:** Mangosteen pericarp powder (MPP) supplementation ameliorated antioxidant enzyme activities, reduced glutathione (GSH) and decreased malondialdehyde (MDA) contents of prostatic tissues in prostatic hyperplasia-rats at the end of the experiment.

	C	P	PL	PH
MDA (nmole/mg protein)	518.6 ± 28.1	884.2 ± 90.4*^b^	411.3 ± 62.4^a^	368.3 ± 56.5^a^
GSH (nmole/mg protein)	4.35 ± 0.50	1.94 ± 0.39*	3.16 ± 0.66	4.01 ± 0.33
SOD (mU/mg protein)	81.11 ± 5.49	77.80 ± 8.92	97.46 ± 16.48	93.04 ± 8.74
GPx (mU/mg protein)	84.3 ± 1.3	43.0 ± 5.7*^a^	84.4 ± 12.9^b^	92.6 ± 17.9^b^
GRd (mU/mg protein)	85.2 ± 15.6	22.9 ± 6.8*	27.4 ± 8.8	37.6 ± 12.6
CAT (mU/mg protein)	44.46 ± 7.04	10.29 ± 2.68*	21.37 ± 8.41	29.71 ± 3.88

^a^Values are presented as the mean ± SEM, *n* = 6; C, control group; P, prostatic hyperplasia-induced group; PL and PH, prostatic hyperplasia-induced and supplemented with low-dose and high-dose MPP groups, respectively; SOD, superoxide dismutase; GPx, glutathione peroxidase; GRd, glutathione reductase; CAT, catalase; *Significantly different between the C and P groups at *p* < 0.05; ^abc^Values in a column with different superscript letters significantly differ at *p* < 0.05 compared to the P group.

## Discussion

In the present study, we evaluated effects of MPP on the development of prostatic hyperplasia in F344 rats in which prostatic hyperplasia was induced by feeding an HFD and injecting DMAB. Results showed increased BW and prostate weight, elevated serum testosterone and DHT levels, prostatic hyperplasia, and overexpression of PCNA, iNOS, and COX-2 in F344 rats with induced prostatic hyperplasia. Moreover, testosterone and DHT levels in group C after 24 weeks of feeding were higher than those in the early stage of the experiment, probably due to aging. These results were consistent with previous research^1,12,17,18^. BPH is caused by the overgrowth of prostatic epithelial and stromal cells. An increased prostate weight, due to prostatic enlargement, is a vital marker of BPH. In this study, the prostate weight of the P group was significantly higher compared to that of the C group (Table 1). Cai *et al*.^28^ reported that after SD rats were fed an HFD (32.2% of total kcal from fat) for 12 weeks, the prostate weight significantly increased, and testosterone and DHT levels were higher than those of rats fed a low-fat diet (3.6% of total kcal from fat). Since androgen plays a vital role in prostate diseases, our results showed that testosterone and DHT levels of the P group were significantly higher compared to those of the C group (Table 3). As the active state of androgen, DHT may induce the growth of prostatic stromal cells^29^. In the present study, H&E staining in the P group showed that atypical cells almost filled the lumen of the ducts. The diameter of the glands was enlarged, and the gland outline was still smooth (Fig. 1). In addition, ki67 protein expression had also increased in the P group compared to the C group (Fig. 1). Sarmento-Cabral *et al*.^30^ reported that ki67 expression increased when nude mice were injected with PC3 (prostate cancer) cells and fed an HFD (60% of total kcal from fat). Those results were further supported by overexpression of PCNA in the P group compared to the C group. However, MPP treatment inhibited PCNA expression (Fig. 2). Using a human prostate cancer cell model, Johnson *et al*.^31^ indicated that due to its structure, α-mangostin, the major xanthone of MPP, inhibited cyclin/cyclin-dependent kinase 4 (CDK4), and treating mice with α-mangostin (100 mg/kg) by oral gavage significantly decreased the average tumor volume in an *in vivo* 22Rv1 tumor xenograft model. Moreover, other research using different cancer cell models also demonstrated that α-mangostin could induce mitochondria-mediated apoptosis through inactivation of the p38 mitogen-activated protein kinase (MAPK) signaling pathway^32,33^. Furthermore, Choi *et al*.^34^ demonstrated that α-mangostin (50 mg/kg) administered for 5 weeks could decrease hepatic steatosis, fat mass accumulation and BW through regulating lipid metabolism via the SIRT1-adenosine monophosphate-activated protein kinase (AMPK) and peroxisome proliferator-activated receptor γ (PPARγ) pathways in mice with HFD-induced obesity. Results of the present study indicated that supplementation with MPP also inhibited increases in BW and prostate weight compared to the C and P groups. We speculate that less prostate weight in MPP group as compared to C group resulted from lower BW of the MPP groups.

Prostatic inflammation is common in prostate diseases^7–9,25^ and it may facilitate cellular proliferation in both benign and malignant conditions. Huang *et al*.^26^ reported that mRNA and protein levels of iNOS were significantly increased in the prostate cancer and BPH with histological-prostatitis groups compared to the BPH group, which play important roles in the development and progression of prostate cancer. Furthermore, Bieniek *et al*.^27^ indicated that treatment with the COX-2 inhibitors, celecoxib and CAY10404, or knockdown of COX-2 significantly inhibited prostate cancer cell proliferation. In the present study, MPP decreased protein expressions of iNOS and COX‐2 (Fig. 2). Chen *et al*.^23^ showed that α-mangostin, a major phenolic compound in the mangosteen pericarp, inhibits protein expression of iNOS and the production of nitric oxide and prostaglandin E_2_ in lipopolysaccharide-stimulated RAW 264.7 cells.

Malondialdehyde (MDA), a marker of lipid peroxidation, is related to oxidative stress^35^. Merendino *et al*.^36^ reported that serum MDA levels were an index of inflammation and oxidative stress in BPH patients, and there was a positive correlation between prostate-specific antigen (a marker of prostatic hyperplasia and prostate cancer) and MDA levels. In the present study, results showed significantly higher MDA levels in prostate tissues of rats with prostatic hyperplasia, but MPP consumption decreased the MDA contents. We speculated that a decrease in MDA was due to the scavenging of free radicals by MPP compounds, and to promoting activities of antioxidant enzymes in prostate tissues. In a previous study, mangosteen pericarp extract supplementation for 11 weeks produced significantly decreased plasma MDA levels and increased activities of superoxide dismutase (SOD), GPx, GRd, and CAT in rats fed an HFD^37^. Fang *et al*.^38^ demonstrated that α-mangostin treatment resulted in increases in SOD and GPx activities and the GSH content of the retina both *in vivo* and *in vitro*. Nelli *et al*.^39^ indicated that α-mangostin treatment showed a noteworthy recovery in testicular and epididymal SOD, catalase, and GPx activities and a decreased MDA level in rats with streptozotocin-induced diabetes. Because there is scant research about ME or α-mangostin upregulating activities of antioxidant enzymes in different tissues, further studies are required in order to elucidate the mechanism.

Mitochondrial complex enzyme activities, including of NCCR, SCCR, and CCO, separately represent electron transport reactions between complexes I and III, and complexes II, III, and IV. Decreases in these complex enzyme activities indicate a decline in mitochondrial ATP synthesis function and greater opportunities for electron leakage and ROS generation. Results of this study revealed that MPP consumption increased NCCR, SCCR, and CCO activities of prostate tissues in rats with induced prostatic hyperplasia. It was reported that isoproterenol can induce a rat myocardial infarction, and after feeding 200 mg/kg BW/day of α-mangostin, NADH dehydrogenase, succinate dehydrogenase, and CCO significantly increased^40^. Thus, MPP might improve mitochondrial function of the prostate in rats with prostatic hyperplasia. This is the first demonstration of the potential utility of MMP in alleviating the abnormal progression of prostatic hyperplasia. Although, MMP contains a substantial amount of α-mangostin, further studies are required in order to elucidate the mechanism of these antiproliferative effects.

## Conclusions

In summary, results of this study suggest that MPP can be used to retard the abnormal progression of prostatic hyperplasia because of its antiproliferative effect, via decreased serum testosterone and DHT levels, and improvements in lipid peroxidation, inflammation, and mitochondrial function of prostate tissues. However, the underlying mechanisms still need to be studied in future research.

## Materials and Methods

### Chemicals and reagents

Dried mangosteen pericarp, containing 4.6% xanthones, was purchased from First Canned Food (Thailand). Dried mangosteen pericarp was ground and sifted through a stainless steel sieve (80 mesh). Dimethyl sulfoxide (DMSO) and 3,2′-dimethyl-4-aminobiphenyl (DMAB) were purchased from Sigma-Aldrich (St. Louis, MO, USA). The dihydrotestosterone (DHT) enzyme-linked immunosorbent assay (ELISA) kit (IB59116) was purchased from Immuno-Biological Laboratories (IBL-America, USA).

### Animals and experimental procedures

Five-week-old male F344 rats (*n* = 24) were purchased from the National Laboratory Animal Center (Taiwan). Animals were housed in an air-conditioned room maintained at 23 ± 2 °C with a relative humidity of 50%~60% and an alternating 12-h light/dark cycle. After 1 week of adaptation, all rats were randomly divided into four groups, including a control group (C), prostatic hyperplasia-induced group (P), prostatic hyperplasia-induced with low-dose MPP group (PL), and prostatic hyperplasia-induced with high-dose MPP group (PH), before the experiment began. The C group was given weekly intraperitoneal (IP) injections of DMSO for 10 weeks, and simultaneously fed a normal diet (AIN 93 G) for 24 weeks. The P, PL, and PH groups were given weekly IP injections of DMAB dissolved in DMSO (25 mg/kg BW) for 10 weeks and simultaneously fed an HFD (50.9% of total kcal from fat) for 24 weeks. The PL and PH groups were separately supplemented with 2.5% and 5% (w/w) MPP in the HFD (Supplementary Table 1). MPP was rich in 68% (w/w) dietary fiber. In order to avoid differences in fiber contents among the diets, the cellulose weight of the treatment diets was respectively adjusted to 34 and 17 g/kg of diet in the PL and PH groups (Supplementary Table 1). Each group contained six rats. Rats in each group were respectively provided with the experimental diet and water *ad libitum*. After being anesthetized with isoflurane gas, blood samples were collected from the tail vein for analysis of serum biochemical parameters at the beginning of the experiment. After 24 weeks of feeding, animals were anesthetized and sacrificed, blood and prostate samples were obtained for further analysis. All animal experimental procedures complied with published guidelines^41,42^ and were approved by the Institutional Animal Care and Use Committee of Taipei Medical University (Taipei, Taiwan; IACUC approval no: LAC-2017-0125).

### Serum biochemical assays

Concentrations of TC, TGs, LDL-C, and HDL-C were measured with an automatic analyzer (Toshiba-2000FR, Japan). The testosterone level was measured by an electrochemiluminescence immunoassay using an automatic analyzer (Roche Cobas^®^e411, USA). The DHT concentration was detected using a DHT ELISA kit according to the manufacturer’s instructions.

### Prostatic histology

Prostate tissues were fixed overnight at room temperature in 10% formaldehyde, dehydrated in 95% ethanol, and then cleared in xylene before being embedded in paraffin. Thick sections (8 μm) were stained with hematoxylin & eosin (H&E), and immunohistochemistry of the ki67 protein was carried out using an anti-ki67 antibody (ab15580; Abcam, USA) and examined with a microscope (Leica ICC50 HD, Germany) (original magnification 100×). Expression of the ki67 protein is strongly associated with tumor cell proliferation and growth, and it is widely used in routine pathological investigations as a proliferation marker. The prognostic value of ki67 has been investigated in many studies with its potential as a reliable marker having been shown in cancers of the lung, soft tissue, breast, and prostate.

### Analysis of prostatic antioxidant enzyme activities and oxidative damage

Prostatic tissue (0.1 g) was homogenized in 0.5 mL buffer (20 M Tris-base, 7 mM NaCl, 1% Triton X-100 at pH 7.2, and 0.1% protease inhibitor) using a homogenizer. Homogenates were centrifuged at 3000 × *g* for 15 min at 4 °C. Protein contents of tissue samples were measured with a Pierce^®^ BCA protein assay kit (Thermo Fisher Scientific, USA) prior to analyzing antioxidant enzyme activities and malondialdehyde (MDA) levels. The activities of antioxidant enzymes, including SOD, CAT, GRd, and GPx, as well as the prostatic GSH content were quantified using commercial kits (Randox Laboratories, UK) according to the manufacturer’s protocols. The level of MDA, a marker of oxidative damage from lipid peroxidation, was evaluated in prostatic tissues using a thiobarbituric acid-reactive substance (TBARS) assay kit (Cayman Chemical, USA).

### Activity analysis of prostatic mitochondrial complex enzymes

Protein contents of tissue samples were measured using a Pierce^®^ BCA protein assay kit prior to analyzing prostatic mitochondrial complex enzyme activities. Activities of mitochondrial complex enzymes were measured according to methods in previous studies, with slight modifications^43,44^. In the assay of NCCR activity, 180 μL of a test solution (1 mM NADH, 1.5 mM potassium cyanide, and 50 mM potassium phosphate buffer; pH 7.4) was added to 10 μg of prostatic mitochondrion extract and incubated at 37 °C for 2 min. Then, 0.5 mM oxidized cytochrome *c* (20 μL) was added. NCCR activity was measured by monitoring the kinetic absorbance at 550 nm every minute for 5 min using a microplate reader (VERSA^®^max, Molecular Devices, USA), and the rate of absorbance alterations was calculated. In the SCCR activity assay, 180 μL of a test solution (25 mM succinate, 1.5 mM potassium cyanide, and 50 mM potassium phosphate buffer; pH 7.4) was mixed with 10 μg prostatic mitochondrion extract and incubated at 37 °C for 2 min. Then, 0.5 mM oxidized cytochrome *c* (20 μL) was added. The kinetic absorbance of SCCR activity was measured at 550 nm for 5 min.

In the CCO activity assay, 200 μL of a test solution (45 μM reduced cytochrome *c* and 50 mM potassium phosphate buffer; pH 7.4) was mixed with 10 μg prostatic mitochondrion extract and incubated at 37 °C for 2 min. As a stop solution, 0.25 M potassium ferricyanide (20 μL) was added. The kinetic absorbance of CCO activity was measured at 550 nm for 5 min.

### Western blot and protein expression analyses

Prostatic tissue (0.1 g) was homogenized in 0.5 mL RIPA buffer (20 mM Tris-HCl, 150 mM NaCl, 1 mM EDTA, 1% Triton-X100, 1% sodium deoxycholate, 0.1% sodium dodecylsulfate, and 0.01% proteinase inhibitor) using a homogenizer (Qiagen^®^ Tissuelyser II, Germany). Homogenates were centrifuged at 13,000 × *g* for 5 min at 4 °C, and then the supernatants were collected. Protein contents of the supernatants were measured prior to analyzing target proteins. Following electrophoretic separation with a 10% sodium dodecylsulfate (SDS) gel, the resolved proteins were electrophoretically transferred to a polyvinylidene difluoride (PVDF) membrane. The following antibodies were used: anti‐PCNA (307901; BioLegend, USA), anti‐iNOS (ab15323; Abcam), anti‐COX‐2 (160112; Cayman Chemical, USA), anti-β-actin (Sigma-Aldrich, USA), goat anti-rabbit horseradish peroxidase (HRP; ab205718; Abcam), and goat anti-mouse HRP (ab205719; Abcam). The bound antibodies were visualized using an enhanced chemiluminescence (ECL) kit (PerkinElmer, USA). The PVDF membranes were first incubated in primary antibody TBST solution overnight at 4 °C, and then probed with HRP-conjugated secondary antibodies for 1 h at room temperature. Signals were analyzed using a UVP^®^ digital imaging system (Analytik Jena US LLC, USA).

### Statistical analysis

Data are expressed as the mean ± standard error of the mean (SEM). Statistical analyses were performed using SPSS statistical software (vers. 19.0; SPSS, USA). Mean differences between parameters were analyzed by a one-way analysis of variance (ANOVA) with Duncan’s multiple-range test for post-hoc comparisons, and a *p* value < 0.05 was considered statistically significant.

## Supplementary information

supplementary information.
