# Supplementary material for: Mangosteen pericarp components alleviate progression of prostatic hyperplasia and mitochondrial dysfunction in rats
Source: Sci Rep. 2020 Jan 15;10:322. doi: 10.1038/s41598-019-56970-2 (PMC6962454; doi:10.1038/s41598-019-56970-2)
Supplement: Supplementary file 1 — supplementary information. [file 41598_2019_56970_MOESM1_ESM.pdf]

Mangosteen pericarp components alleviate progression of prostatic hyperplasia and mitochondrial dysfunction in rats

Hui-Hsuan Tsai[1], Chia-Wen Chen[1], Pei-Ling Yu[1], Yu-Ling Lin[1], Rong-Hong Hsieh[1]

[1]*School of Nutrition and Health Sciences, College of Nutrition, Taipei Medical University, Taipei, Taiwan*

**Corresponding author: hsiehrh@tmu.edu.tw*

Co-authors:

[1] Hui-Hsuan Tsai, email: ma07105009@tmu.edu.tw

[2] Chia-Wen Chen, email: d301091008@tmu.edu.tw

[3] Pei-Ling Yu, email: ga56105008@tmu.edu.tw

[4] Yu-Ling Lin, email: ma07105020@tmu.edu.tw

School of Nutrition and Health Sciences, College of Nutrition, Taipei Medical University, 250 Wu-Hsing St., Taipei 11031, Taiwan.

Office phone: +886-2-27361661 ext. 6566

Corresponding author:

Rong-Hong Hsieh

Email: hsiehrh@tmu.edu.tw

School of Nutrition and Health Sciences, College of Nutrition, Taipei Medical University, 250 Wu-Hsing St., Taipei 11031, Taiwan.

Office phone: +886-2-27361661 ext. 6557

Supplementary table 1. The components of experimental groups

**Component** (g/kg)	**C**	**P**	**PL**	**PH**
Casein	200	200	200	200
L-Cystine	3	3	3	3
Corn starch	530	315	315	315
Sucrose	100	100	100	100
Cellulose	50	50	34	17
Soybean oil	70	70	70	70
Lard	0	205	205	205
AIN-93 Mineral Mix	35	35	35	35
AIN-93 Vitamin Mix	10	10	10	10
Cholesterol	10	10	10	10
Choline bitartrate	2.5	2.5	2.5	2.5

	**C**	**P, PL, PH**
**Selected Nutrition Information**	% of total energy	% of total energy
Carbohydrate	63.6	33
Fat	15.9	50.9
Protein	20.5	16.1

C: Control

P: prostatic hyperplasia-induced

PL: prostatic hyperplasia-induced with low-dose MPP

PH: prostatic hyperplasia-induced with high-dose MPP

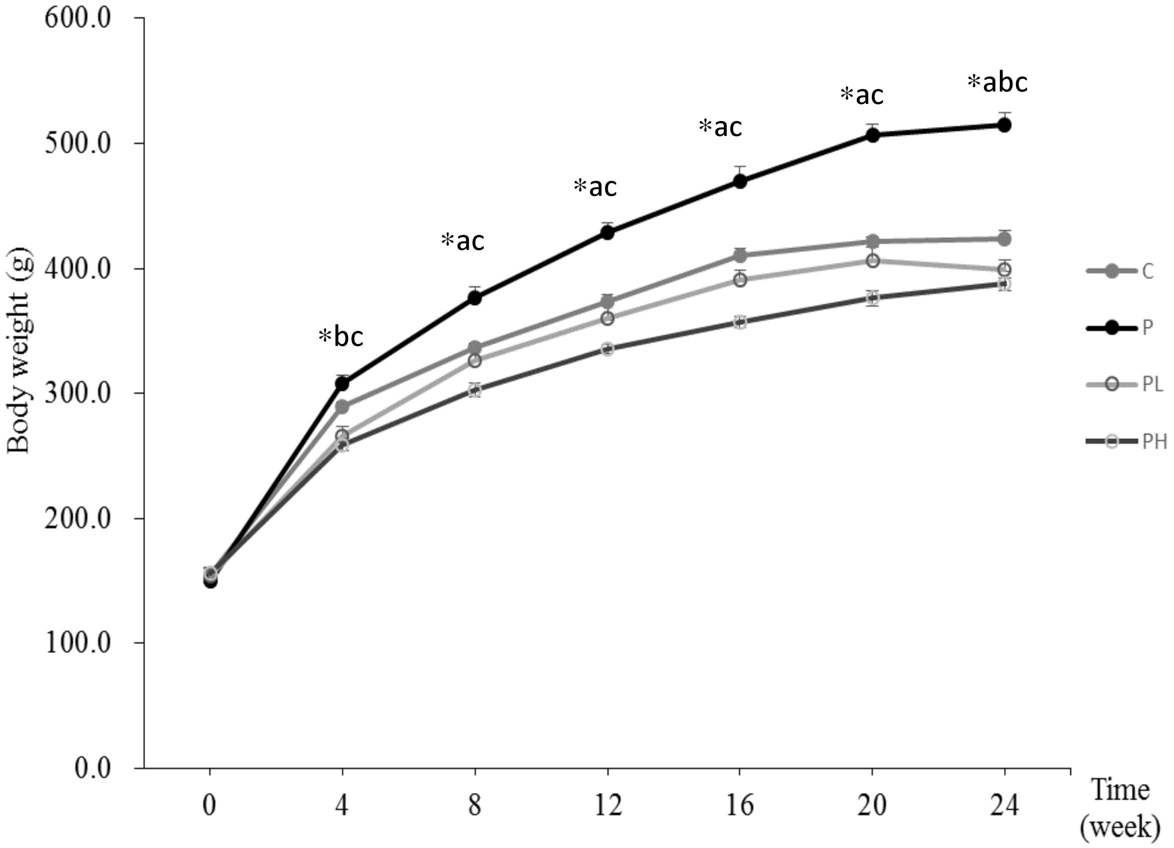

Supplementary figure 1. MPP supplementation decreased the body weight gain of experimental groups. C, control diet group; P, prostatic hyperplasia-induced group; PL and PH, prostatic hyperplasia-induced and supplemented with low-dose and high-dose mangosteen pericarp powder (MPP) groups, respectively. Plots are presented as the mean ± SEM (n=6); * Significantly different between the P, PL and PH groups ($p < 0.05$). Plots at the same time point with the letter “a” significantly differ between the C and P groups; “b” indicates significantly differ between the C and PL groups; “c” indicates significantly differ between the C and PH groups. Body weight of the PL and PH groups did not have significant difference during the experimental period.

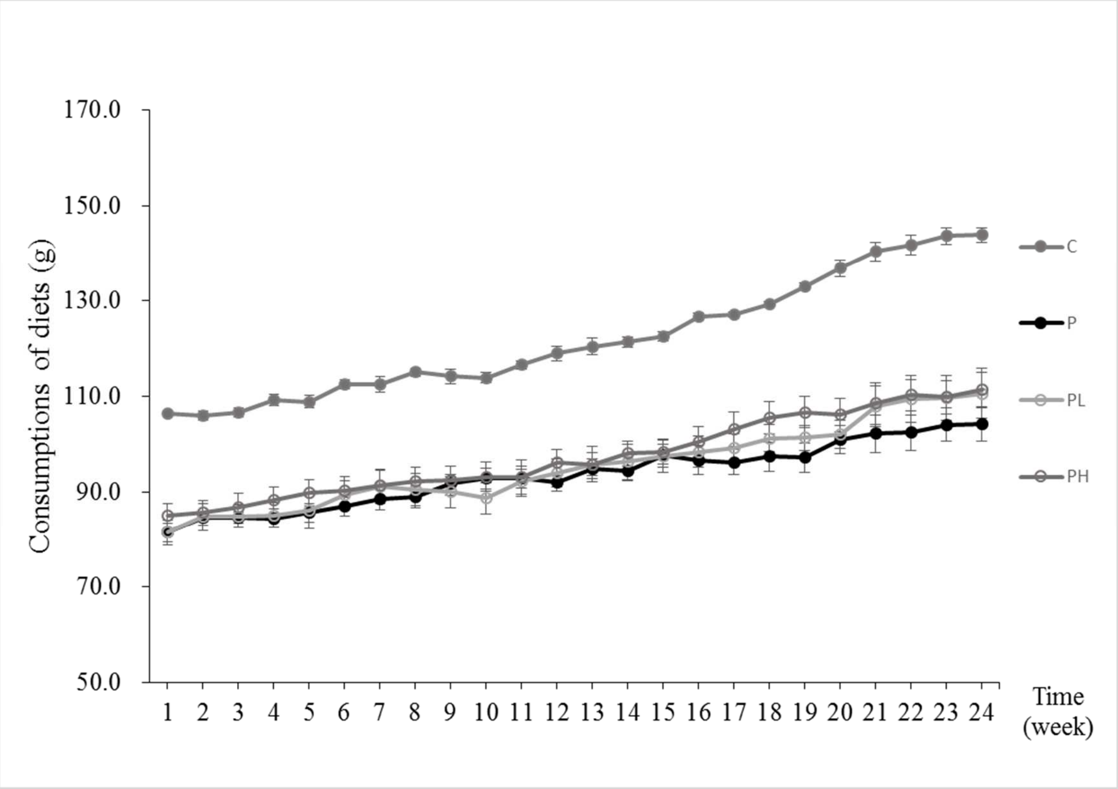

Supplementary figure 2. Consumptions of the diets per week in all groups. Plots are presented as the mean ± SEM (*n*=6); C, control diet group; P, prostatic hyperplasia-induced group; PL and PH, prostatic hyperplasia-induced and supplemented with low-dose and high-dose mangosteen pericarp powder groups, respectively. Diet consumptions per week of the prostatic hyperplasia-induced groups all had significant difference compared to the C group, but did not have significant difference between prostatic hyperplasia-induced groups during the experimental period.
